# Adjuvant and neoadjuvant therapy for gastric cancer using epirubicin/cisplatin/5-fluorouracil (ECF) and alternative regimens before and after chemoradiation

**DOI:** 10.1038/sj.bjc.6601311

**Published:** 2003-10-14

**Authors:** T Leong, M Michael, K Foo, A Thompson, D Lim Joon, L Weih, S Ngan, R Thomas, J Zalcberg

**Affiliations:** 1 Peter MacCallum Cancer Centre, St Andrews Place, Melbourne, Victoria 3002, Australia

**Keywords:** ECF, adjuvant therapy, chemoradiation, gastric cancer

## Abstract

Chemoradiation is now used more commonly for gastric cancer following publication of the US Intergroup trial results that demonstrate an advantage to adjuvant postoperative chemoradiotherapy. However, there remain concerns regarding the toxicity of this treatment, the optimal chemotherapy regimen and the optimal method of radiotherapy delivery. In this prospective study, we evaluated the toxicity and feasibility of an alternative chemoradiation regimen to that used in the Intergroup trial. A total of 26 patients with adenocarcinoma of the stomach were treated with 3D-conformal radiation therapy to a dose of 45 Gy in 25 fractions with concurrent continuous infusional 5-fluorouracil (5-FU). The majority of patients received epirubicin, cisplatin and 5-FU (ECF) as the systemic component given before and after concurrent chemoradiation. The overall rates of observed grade 3 and 4 toxicities were 38 and 15%, respectively. GIT grade 3 toxicity was observed in 19% of patients, while haematologic grade 3 and 4 toxicities were observed in 23%. Our results suggest that this adjuvant regimen can be delivered safely and with acceptable toxicity. This regimen forms the basis of several new studies being developed for postoperative adjuvant therapy of gastric cancer.

Carcinoma of the stomach remains a major cause of cancer-related death in most Western countries. Surgery is the only proven effective therapy, but overall 5-year survival rates remain low after resection. Patterns of failure data from Western series document that approximately 60% of those with positive lymph nodes or extension of the primary tumor through the serosa fail locoregionally (Gunderson and Sosin, 1982; Landry *et al*, 1990). The recently reported Gastric Surgical Adjuvant Trial (INT0116) has established combined chemoradiotherapy as an integral component of standard adjuvant therapy for high-risk, completely resected adenocarcinoma of the stomach (Macdonald *et al*, 2001). There was a major survival advantage to the use of combined modality therapy postoperatively, with 3-year survival improving from 41 to 50%. These data support the undertaking of further studies to build on the results of the Intergroup study, as there remain several concerns among both medical and radiation oncologists regarding implementation of this treatment.

The first relates to the toxicity associated with the treatment. The combined modality regimen in this programme was associated with considerable toxicity, with grade 3 and 4 toxicities occurring in 41 and 32% of cases, respectively (Macdonald *et al*, 2001).

The second area of concern relates to the optimal chemotherapy regimen. The Intergroup study employed bolus 5-fluorouracil (5-FU) and leucovorin delivered before, during and after radiation. This regimen was chosen because it represented the standard of care at the time the trial was developed in the early 1990s. However, many medical oncologists feel that this regimen is now outdated and that there are more active regimens available for gastric cancer. The failure pattern data from INT0116 suggest a minimal effect of 5-FU/leucovorin on regional and distant failure (Macdonald *et al*, 2001). The high recurrence rate, even in the chemoradiation arm, clearly indicates the need for improved systemic therapies.

The last area of concern relates to the radiotherapy planning and treatment techniques employed in INT0116. Radiotherapy fields were designed using conventional simulation with minimal use of CT planning to define the clinical target volume, and all patients were treated with simple parallel-opposed anterior and posterior field arrangements (Smalley *et al*, 2002). However, many radiation oncologists are reluctant to treat such large abdominal volumes with anterior and posterior fields due to concerns about normal tissue toxicity, particularly in relation to the kidneys and spinal cord. Recent computer-planning developments in radiotherapy techniques offer major advantages over the traditional opposed-field approaches devised in the 1960s, with the potential to reduce normal tissue toxicity (Dobelbower *et al*, 1980; Chu *et al*, 1992).

Following the initial reporting of INT0116 results (ASCO 2000), we began treating patients using an alternative regimen of chemoradiation for gastric cancer, employing a more current chemotherapy combination of epirubicin, cisplatin and 5-fluorouracil (ECF), given before and after concurrent chemoradiation. The chemoradiation component combined modern conformal radiotherapy techniques with continuous infusional 5-FU at a standard dose of 225 mg m^−2^ day^−1^. As this regimen represents current standard practice at our institution, informed consent and Ethics Committee approval were not required. Patient and treatment data were collected prospectively as part of a pilot study to evaluate the toxicity and feasibility of this regimen.

## MATERIALS AND METHODS

### Patients

The study population consisted of two groups of patients: (1) Those with histologically confirmed adenocarcinoma of the stomach or gastro-oesophageal junction, who had undergone a complete R0 resection with negative margins. Patients in this group were eligible for postoperative adjuvant therapy if they had: tumour stage T3–4 and/or N1–2; performance status of 2 or lower according to the criteria of the Eastern Cooperative Oncology Group (ECOG) and adequate oral nutrition (caloric intake of 1500 cal per day). (2) Those with locally advanced, histologically confirmed adenocarcinoma of the stomach or gastro-oesophageal junction not considered suitable for surgical resection based on clinical evaluation of tumour size, invasion of adjacent structures and advanced locoregional node involvement. Two patients who were considered unsuitable for surgery based on medical comorbidity were also eligible. Patients in this group were eligible for definitive chemoradiation if they had: no evidence of distant metastases; ECOG performance status 2 or lower and adequate nutritional status. Several patients who achieved a good response to chemoradiation underwent subsequent surgical resection.

### Treatment

The chemoradiation regimen was the same for both patient groups. The majority of patients received one cycle of ECF chemotherapy (epirubicin 50 mg m^−2^ IV day 1, cisplatin 60 mg m^−2^ IV day 1 and 5-FU 200 mg m^−2^ day^−1^ IV 21-day continuous infusion) initiated on day 1, and this was followed by chemoradiotherapy beginning 28 days after the start of the initial cycle of chemotherapy. Chemoradiotherapy consisted of 45 Gy of radiation at 1.8 Gy per day, 5 days per week for 5 weeks, with concurrent continuous infusion 5-FU (225 mg m^−2^ day^−1^, 7 days per week, throughout the entire period of radiotherapy). Continuous infusion 5-FU was delivered by ambulatory pump via a central venous access device with 1 mg warfarin prophylaxis. All patients received 5HT3 antagonists on day 1 of ECF and continued for 2 days after. At 1 month after the completion of radiotherapy, two further cycles of ECF chemotherapy were given. A small number of patients received alternative fluorouracil-based regimens before and after radiation, most commonly fluorouracil and leucovorin (fluorouracil 425 mg m^−2^ day^−1^ and leucovorin 20 mg m^−2^ day^−1^ for 5 days), as delivered in INT0116.

Radiotherapy generally followed the recommendations outlined in INT0116 (Macdonald *et al*, 2001). The 45 Gy of radiation was delivered in 25 fractions, five days per week, to the tumour bed, anastomoses and stumps, and regional lymphatics. The design of the radiation treatment fields for postoperative treatment was individualised depending upon the extent and location of the primary tumour and involved lymph nodes, and the type of surgery performed. For definitive chemoradiation, the radiation treatment fields were the same as for postoperative treatment, except that there are no anastomoses that need to be covered. Lymph node stations included in the radiation fields included perigastric, coeliac, splenic hilar, suprapancreatic, porta hepatis, pancreaticoduodenal and local para-aortic nodes. In patients with tumours confined to the proximal one-third of the stomach or gastro-oesophageal junction with limited lymphatic invasion, treatment of the pancreaticoduodenal nodes was omitted. Similarly, treatment of the splenic hilar nodes was omitted in patients with tumours of the lower third of the stomach or antrum. The clinical target volume (CTV) was defined in all patients using CT planning, and all were treated using a standardised 3D conformal technique that consisted of a ‘split-field’, monoisocentric arrangement employing six radiation fields. Radiation was delivered using 6–18 MV photons. Dose–volume histograms (DVHs) were recorded for the kidneys, liver and spinal cord in all patients. Baseline renal scans were performed to assess differential renal function and glomerular filtration rate (GFR) prior to commencing radiotherapy.

### Patient follow-up

All patients were reviewed weekly during treatment, and two weekly post-treatment for 6 weeks with physical examination, toxicity assessment and full blood count and biochemistry. Acute toxicities were graded using the NCI common toxicity criteria. Late effects were graded according to RTOG/EORTC late effects criteria. For the purposes of comparison with the Intergroup study, acute toxicities have been presented using the criteria of the Southwest Oncology Group (SWOG). Follow-up of both groups occurred at 3-month intervals for 2 years, then at 6-month intervals for 3 years, and yearly thereafter. The follow-up consisted of physical examination, full blood count and biochemistry. CT and PET scanning was only performed if clinically indicated. Patients receiving definitive chemoradiation usually underwent repeat gastroscopy to assess the response. The site and date of the first relapse and the date of death, if the patient died, were recorded.

## RESULTS

### Patient characteristics

Between July 2000 and May 2002, 26 patients were treated (Table 1

**Table 1:** Patient characteristics

**Characteristic**	
No. of patients	26
	
*Age (years)*	
Median	60
Range	34–82
	
*Sex*	
Male	21
Female	5
	
*Treatment*	
Postoperative	18
Definitive	8
	
*Nodal dissection*	
D2	1
D1	6
<D1	11
	
*T stage (postoperative patients)*	
T2	4
T3	13
T4	1
	
*Location of primary tumour*	
G–O junction/cardia	10
Body	9
Antrum	7
	
*Chemotherapy*	
ECF	17
CI 5-FU during RT	24

G–O, gastro-oesophageal; CI, continuous infusional; RT, radiotherapy.

). The majority received postoperative adjuvant treatment following complete surgical resection. One patient underwent a formal D2 lymph node dissection, six patients underwent D1 nodal dissections and 11 patients underwent less than D1 nodal dissections. The 18 patients who underwent initial surgical resection were staged according to 2002 UICC criteria: Stage II-2, Stage IIIA-10, Stage IIIB-4 and Stage IV-2. The majority had T3 tumours and all had involved lymph nodes. Of the eight patients who were treated with definitive chemoradiation, three underwent subsequent surgical resection. The location of the primary tumour within the stomach showed a slight predominance of cardiac and gastro-oesophageal junction tumours.

**Figure 1:**
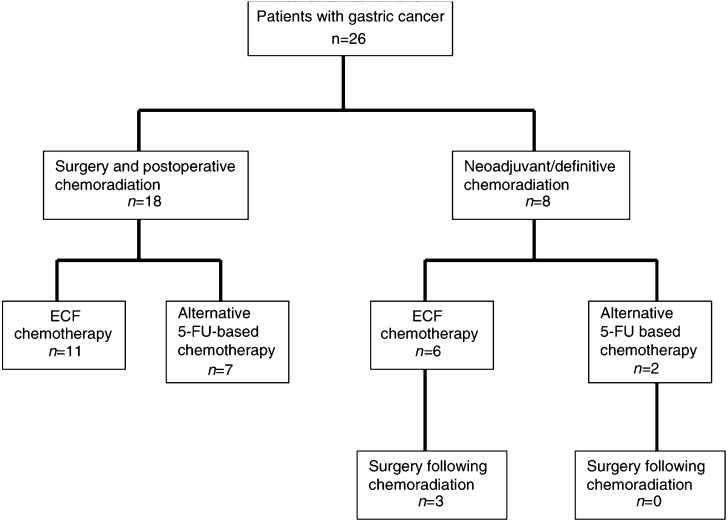
Flow diagram showing treatment delivered.

There was some variation in the chemotherapy regimens delivered (Table 1). Since the chemotherapy that was delivered concurrently with radiation was usually administered by medical oncologists from our institution, over 90% of the patients received continuous infusional 5-FU with radiotherapy. Two patients received fluorouracil/leucovorin during radiation, as given in INT0116. However, the chemotherapy that was delivered before and after radiation was, in some patients, administered by medical oncologists from outside institutions, some of whom did not use ECF chemotherapy. Overall, approximately 70% of patients received ECF, while the remainder received alternative fluorouracil-based regimens, usually fluorouracil/leucovorin. All treatments received by all 26 patients are summarised in Figure 1

.

### Treatment toxicity

Of the 26 patients who commenced chemoradiation, 21 (81%) completed treatment as planned. One patient receiving definitive chemoradiation failed to complete his continuous infusional 5-FU during radiotherapy. This patient developed a pulmonary embolus in week 5 of radiotherapy and his chemotherapy was ceased. The last two cycles of ECF were not given. Two further patients receiving definitive chemoradiation failed to complete their postchemoradiation ECF chemotherapy: one underwent surgery following a good partial response to one cycle of ECF and combined chemoradiation, and the other was not given the last two cycles of ECF due to grade 3 nausea and mucositis. One patient receiving postoperative treatment refused the last cycle of ECF chemotherapy because of persistent nausea. Another postoperative patient (receiving the INT0116 regimen) required a radiotherapy treatment break (of 1 week) for nausea and vomiting. All other patients completed their radiotherapy as planned, without treatment breaks.

The major acute toxic effects attributable to the adjuvant regimen (both combined chemoradiation and chemotherapy without radiation) were defined as those of grade 3 or higher, and are summarised in Table 2

**Table 2:** Acute toxicity

	**All patients (*n*=26)**	**All patients receiving ECF (*n*=17)**	**All postoperative patients (*n*=18)**	**All definitively treated patients (*n*=8)**	**Postoperative patients receiving ECF (*n*=11)**
	**% (no.)**				
Overall grade 3	38 (10)	41 (7)	28 (5)	63 (5)	27 (3)
Overall grade 4	15 (4)	24 (4)	11 (2)	25 (2)	18 (2)
GIT grade 3	19 (5)	18 (3)	17 (3)	25 (2)	9 (1)
Haematologic grade 3 and 4	23 (6)	29 (5)	22 (4)	25 (2)	27 (3)

Major toxic effects were defined as those of grade 3 or higher. Toxic effects have been presented according to SWOG criteria. Data are presented for: all patients who received chemoradiotherapy; patients who received ECF; patients treated postoperatively; patients treated with definitive chemoradiation; and postoperative patients who received ECF. ECF=epirubicin, cisplatin and 5-fluorouracil; GIT=gastrointestinal.

. The overall rate of grade 3 toxicity was 38%. Grade 4 toxicity occurred in only four patients (15%); two developed neutropenia without sepsis, one developed severe fatigue requiring hospitalisation, and one developed a pulmonary embolus which was not necessarily treatment related. For those patients who received ECF chemotherapy, the rates of grade 3 and 4 toxicities were 41 and 24%, respectively. The most commonly reported toxic effects were gastrointestinal (GIT) and haematologic. The overall rate of grade 3 GIT toxicity was 19%, with no patients experiencing grade 4 GIT toxic effects. Gastrointestinal toxicity consisted mainly of nausea that occurred predominantly during concurrent chemoradiation. Haematologic grade 3 and 4 toxicities were reported in 23% of patients and consisted mainly of leukopenia occurring during chemotherapy without radiation. For patients receiving ECF chemotherapy, the corresponding rates of grade 3 GIT and grade 3/4 haematologic toxicities were 18 and 29%, respectively. The toxicity rates for patients receiving definitive chemoradiation *vs* postoperative chemoradiation are also shown in Table 2, as well as those for postoperative patients who were treated with ECF chemotherapy. There were no treatment-related deaths.

Late radiation toxicity has been observed in only one patient who developed RTOG grade 3 enteritis. At 4 months after completing chemoradiation, this patient developed persistent melena that necessitated repeated blood transfusions. Gastroscopy and enteroscopy revealed inflammatory changes affecting the mucosa of the duodenum and proximal jejunum. Laparoscopy demonstrated oedema of the duodenum and proximal jejunum, but normal-appearing small bowel elsewhere. Abnormalities were also noted on CT, which showed thickening of the small bowel wall involving the duodenum. The distribution of these small bowel abnormalities was found to correspond to radiation fields that were employed to treat the infrapyloric and pancreaticoduodenal lymph nodes. The patient's symptoms resolved after approximately 3 months, without any specific treatment.

### Disease relapse

**Figure 2:**
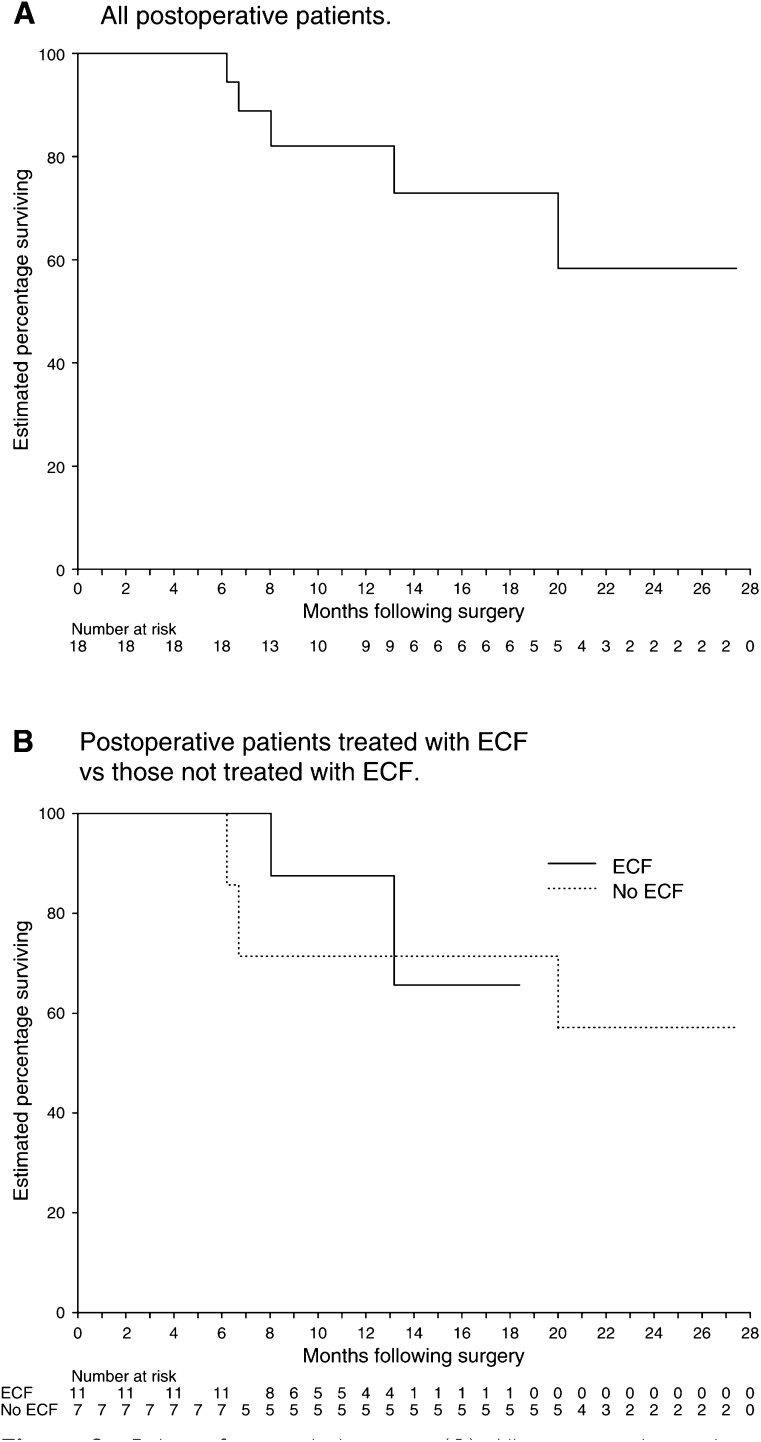
Relapse-free survival among: (**A**) All postoperative patients; and (**B**) Postoperative patients treated with ECF *vs* those not treated with ECF. Five postoperative patients have relapsed and three have died. Relapse-free survival was defined as the interval between date of surgery and date of first relapse or death (if death preceded relapse).

The median follow-up period for all 26 patients is 12 months. The site of first relapse was recorded for the 18 patients who were treated postoperatively. Of these 18 patients, 11 were treated with ECF chemotherapy and seven were treated with alternative fluorouracil-based regimens. Two of the 11 patients who received ECF developed recurrence: one with peritoneal carcinomatosis, and one with both peritoneal carcinomatosis and liver metastases. Both patients have died following recurrence. Three of the seven patients who received non-ECF chemotherapy developed recurrence: one with locoregional disease, one with liver metastases and one with peritoneal carcinomatosis, who subsequently died following recurrence. Using Kaplan–Meier estimates, the 1-year relapse-free survival for all 18 postoperative patients is 82% (standard error 9%) (Figure 2a

). Estimated 1-year relapse-free survival for the ECF group is 87% (standard error 11%), and for the group that did not receive ECF it is 71% (standard error 17%) (Figure 2b).

Three patients who were treated with definitive chemoradiation for initially unresectable disease subsequently underwent surgery following a favourable response to treatment. One patient with a locally advanced tumour of the cardia and extensive regional lymphadenopathy obtained a complete response both at the primary site and involved lymph nodes, as assessed endoscopically (with biopsy) and on follow-up PET scan. He underwent complete surgical resection, but unfortunately developed cerebral metastases 9 months after completion of chemoradiation. The remaining two patients obtained a partial response following chemoradiation and remained alive and disease free 10 and 21 months after complete surgical resection.

## DISCUSSION

Worldwide, large amounts of resources have been expended in the search for an effective adjuvant therapy to reduce the risk of postoperative relapse following surgery for gastric cancer. Postoperative adjuvant chemotherapy for gastric cancer has been thoroughly explored over the past 10–15 years. Several recent meta-analyses have indicated that, even with pooled data, postoperative adjuvant systemic therapy produces only a modest improvement in survival (Earle and Maroun, 1999; Mari *et al*, 2000; Janunger *et al*, 2001). Likewise, adjuvant radiotherapy alone has also failed to demonstrate any survival benefit (Hallissey *et al*, 1994). Recognition of the high locoregional failure rates following surgery has resulted in four separate randomised trials evaluating the role of extended lymph node dissection (Robertson *et al*, 1994; Dent *et al*, 1988; Bonenkamp *et al*, 1999; Cuschieri *et al*, 1999). All the four trials demonstrate a substantial increase in morbidity and, in some series, operative mortality with extended lymph node dissection. None show improvement in overall survival.

The results of the US Intergroup trial, which demonstrate a survival advantage to the use of adjuvant chemoradiotherapy, have changed the standard of care in some parts of the world for the use of both chemotherapy and radiotherapy in the postoperative setting for patients with high-risk disease. Implementation of this treatment has, however, posed several problems with respect to both chemotherapy and radiotherapy. At the same time, it has also provided new opportunities to build on the Intergroup results by trying to improve treatment efficacy and reduce treatment toxicity.

The chemotherapy regimen used in INT0116 was associated with considerable toxicity and some medical oncologists are reluctant to use this regimen given that more active and apparently less toxic combinations are now available. The Intergroup study was initiated over 10 years ago and the chemotherapy that was used was not optimised, but it was adopted since this was a regimen that had been through toxicity studies. An analysis of failure patterns indicates that the improvement noted in INT0116 was entirely due to an improvement in local control with no effect on distant metastases (Macdonald *et al*, 2001). This strongly suggests that the 5-FU/leucovorin combination, as delivered in this study, is producing its effect through radiosensitisation to assist radiation therapy in obtaining local control.

In our study, the majority of patients received ECF both before and after chemoradiation. This regimen was first reported in 1991, with the three drugs being selected on the basis of their single agent activity in upper GIT tumours (Beer *et al*, 1983; Cersosimo and Hong, 1986; Machover *et al*, 1986), and on the synergy demonstrated between 5-FU and cisplatin in preclinical models (Etienne *et al*, 1991). ECF is associated with response rates of approximately 45% in patients with metastatic gastric cancer, although higher response rates are seen when used for locally advanced disease (Findlay *et al*, 1994; Waters *et al*, 1999). We also employed continuous infusional 5-FU delivered concurrently with radiation. Continuous, as opposed to bolus, 5-FU during radiation for other GIT tumours is less toxic and better tolerated, and is proposed to maximise the opportunities for radiosensitisation (O-Connell *et al*, 1994; Poen *et al*, 1998).

The toxicity rates observed in this study compare favourably to those reported in INT0116. The overall rates of grade 3 and 4 acute toxicity for our patients were 38 and 15%, respectively. For those patients receiving ECF, the corresponding rates were 41 and 24%. However, this group includes some patients treated with definitive chemoradiation, who, in general, were less medically fit and had more advanced tumours requiring larger radiation fields. If we exclude these patients, then the rates of grade 3 and 4 toxicities for postoperative patients receiving ECF were 27 and 18%. As described above, GIT and haematologic toxic effects predominated. For the patient groups listed in Table 2, the rates of grade 3 GIT toxicity range from 9 to 25%, while the rates of grade 3/4 haematologic toxicity range from 22 to 29% for the different subgroups. The lower rates of haematologic and overall grade 4 toxicity compared to those reported in INT0116 (54 and 32%, respectively) may reflect the chemotherapy regimen employed, particularly the use of continuous infusional 5-FU, which is known to produce less myelotoxicity compared to bolus 5-FU. Although a number of patients in this study did not receive ECF, the majority (90%) did receive continuous infusional 5-FU during radiation.

A further contributing factor to the favourable toxicity profile, particularly GIT toxicity, may be the use of more conformal radiotherapy techniques compared to the simple anterior and posterior fields used in INT0116. All of our patients were able to complete their radiotherapy as planned, whereas 17% of patients in the Intergroup study did not complete their radiotherapy due to toxic effects. The volume of the small bowel that is included in the radiation fields is reduced by using multiple fields that conform to the high-risk target volume. Very few of our patients experienced any significant vomiting or diarrhoea and the most common acute GIT toxic effect was nausea. A major concern among radiation oncologists is the potential for late toxicity associated with high-dose radiation to the abdomen. By using CT-assisted computer planning and 3D conformal radiation techniques, we have been able to reduce the dose to critical normal structures such as kidneys, spinal cord and small bowel. Comparative dose–volume histograms comparing our multiple-field technique with an anterior/posterior technique demonstrate considerable sparing of these critical structures from the high-dose volume (data not shown).

The short median follow-up precludes any meaningful analysis of relapse and survival patterns. However, our 1-year relapse-free survival rate for postoperative patients compares favourably to that observed for patients in the adjuvant chemoradiation arm of the Intergroup study. Longer follow-up and increased patient numbers are required to compare the outcomes of patients receiving ECF chemotherapy with those receiving alternative fluorouracil-based regimens.

The Intergroup study is often criticised because of the inadequacy of the nodal dissections performed with 54% of patients undergoing less than a D1 lymph node dissection. It has been claimed that the benefits of chemoradiation are only due to the compensation of poor surgery, and that these benefits would not be seen if a D1 or D2 dissection had been performed. However, this is a hypothesis that remains to be proven. To date, the published randomised trials have not shown that more extensive nodal dissections are associated with improved survival. We, therefore, do not have definitive data indicating that, in a multi-institutional study, D2 operations are superior. It is possible that better surgery using a formal D1 or D2 dissection may decrease the need for radiation. On the other hand, it is also possible that when better surgery is combined with chemoradiation, there may be a further improvement in local control as seen in the Intergroup study. The role of radiotherapy in preventing locoregional recurrence for patients who have undergone at least a D1 dissection would therefore be an appropriate question for a future clinical trial.

The preliminary report of the MRC MAGIC trial (ASCO 2003) also supports the use of ECF chemotherapy in the adjuvant and neoadjuvant setting. This trial has shown that perioperative ECF improves progression-free survival in patients with operable gastric cancer. While the survival benefit seen in the US Intergroup trial was mainly due to improved local control from radiotherapy (with no effect on distant metastases), the benefit observed in the MAGIC study was due mainly to a reduction in distant metastases. It would therefore seem logical to combine optimal local treatment with optimal systemic treatment, as we have done in this study.

The emergence of combined chemoradiotherapy as an effective adjuvant treatment with significant benefit provides new opportunities to develop improved treatment by combining more effective systemic therapies with modern conformal techniques of radiation delivery. Our results suggest that an adjuvant regimen employing ECF as the systemic component before and after concurrent chemoradiation with continuous infusional 5-FU can be delivered safely and with acceptable toxicity. This study forms the basis of a new Australasian multicenter trial conducted under the auspices of the Trans-Tasman Radiation Oncology Group (TROG) that will evaluate the feasibility of this regimen on a national scale, and attempt to standardise radiation oncology techniques among centres that have different practices and equipment. An important aspect of this study is the radiation oncology quality control review procedure that will involve ‘real time’ central review of all radiotherapy treatment plans prior to patients commencing treatment. A detailed set of radiotherapy guidelines has been developed to ensure the accuracy and consistency of radiotherapy planning.
